# Substance P Promotes the Proliferation, but Inhibits Differentiation and Mineralization of Osteoblasts from Rats with Spinal Cord Injury via RANKL/OPG System

**DOI:** 10.1371/journal.pone.0165063

**Published:** 2016-10-20

**Authors:** Hai-Juan Liu, Hua Yan, Jun Yan, Hao Li, Liang Chen, Li-Ren Han, Xiao-Fei Yang

**Affiliations:** 1 Department of Endocrinology, Liaocheng People’s Hospital, Shandong, China; 2 Department of Medical Service, Navy General Hospital, Beijing, China; 3 Department of Orthopaedic Surgery, Liaocheng People’s Hospital, Shandong, China; The University of Adelaide, AUSTRALIA

## Abstract

Spinal cord injury (SCI) causes a significant amount of bone loss, which results in osteoporosis (OP). The neuropeptide substance P (SP) and SP receptors may play important roles in the pathogenesis of OP after SCI. To identify the roles of SP in the bone marrow mesenchymal stem cell derived osteoblasts (BMSC-OB) in SCI rats, we investigated the expression of neurokinin-1 receptors (NK1R) in BMSC-OB and the effects of SP on bone formation by development of BMSC-OB cultures. Sixty young male Sprague-Dawley rats were randomized into two groups: SHAM and SCI. The expression of NK1R protein in BMSC-OB was observed using immunohistochemistry and Western blot analysis. The dose- and time-dependent effects of SP on the proliferation, differentiation and mineralization of BMSC-OB and the expression of osteoblastic markers by *in vitro* experiments. The expression of NK1R in BMSC-OB was observed on plasma membranes and in cytoplasm. One week after osteogenic differentiation, the expression of NK1R was significantly increased after SCI at mRNA and protein levels. However, this difference was gradually attenuated at 2 or 3 weeks later. SP have the function to enhance cell proliferation, inhibite cell differentiation and mineralization at a proper concentration and incubation time, and this effect would be inhibited by adding SP or NK1R antagonist. The expression of RANKL/OPG was significantly increased in tibiae after SCI. Similarly, the RANKL/OPG expression in SCI rats was significantly increased when treating with 10^−8^ M SP. SP plays a very important role in the pathogenesis of OP after SCI. The direct effect of SP may lead to increased bone resorption through the RANKL/OPG axis after SCI. In addition, high expression of SP also results in the suppression of osteogenesis in SCI rats. Then, the balance between bone resorption and bone formation was broken and finally osteoporosis occurred.

## Introduction

Osteoporosis (OP), one of common complications caused by spinal cord injury (SCI), manifests rapid bone loss [1–3], bone-ultrastructural degeneration [4], decline of bone-biomechanical property and increases fracture risk [5, 6]. The mechanism of SCI-induced OP is poorly elucidated. Previously, immobilization was generally considered to be the main cause of SCI-induced bone loss [7–9], but current studies suggested that the mechanisms of OP induced by SCI are more complicated and neural lesion itself may be involved in the bone loss [10, 11]. Compared to disuse OP, the levels of bone resorption markers are significantly increased in SCI-induced OP. The sites and rate of bone loss in SCI-induced OP are different from that in disuse OP [12, 13]. Furthermore, functional exercise may contribute to the reversion of bone mass in disuse-induced bone loss, but cannot prevent demineralisation in the SCI-induced bone loss [14]. Therefore, OP after SCI is a complex process including multiple pathogenetic factors, and should not be simply considered disuse OP. Understanding the pathogenesis of SCI-induced OP will be benefit in the consideration of new treatment strategies [15, 16].

According to prior studies, the neural lesion after SCI may play a pivotal role in the pathogenesis of OP by taking a direct role in denervation on bone remodeling, or by disrupting vasoregulation as an indirect role [17]. Neuropeptide SP, a kind of neurotransmitter which can transmit the information of noxious stimulation, is widely distributed in the central and peripheral nervous system and mainly resides in unmyelinated sensory afferent fibers. It can be transported to nerve endings and released through axon reaction. SP and calcitonin gene-related peptide (CGRP) exist simultaneously in peripheral nerves [18]. When the nerve endings are stimulated, the dorsal root ganglia begin to synthesize SP. SP is involved in inflammatory reaction such as vasodilatation and plasma exosmose. Sensory nerve fibers are widely distributed in bone tissue, especially in bone marrow and periosteum [19]. Recent studies have indicated that SP plays a role in bone metabolism through regulating bone formation of osteoblasts and bone resorption of osteoclasts [20].

Our previous study showed that SP-positive nerve fibers were increased in bone tissue below the level of demage in rats with SCI [21]. Does the increased expression of SP have a relationship with SCI-induced OP? And whether the change of biological behaviors of osteoblasts is through the interaction between SP and its receptor Neurokinin-1 (NK1R) remains to be further confirmed. Our previous study has revealed that SP may increase bone formation and osteogenic activity through RANKL/OPG signaling system [22]. The purpose of this study was to investigate the expression of NK1R in BMSC-OB, detect the effects of SP on the biological behaviors (including proliferation, differentiation and mineralization) of BMSC-OB at different concentrations and time points in rats with SCI, and to reveal the mechanism of SP in SCI-induced OP.

## Materials and Methods

### Animals

All animal experiments were performed following The Regulation on animal experimentation of ethics committee of Shandong University. This study was approved by the Animal Care and Use Committee of ethics committee of Shandong University. Rats were purchased from the Experimental Center of Liaocheng People’s Hospital. Sixty male Sprague-Dawley rats (6 weeks) were randomly divided into two groups: sham-operated (SHAM) group (n = 30) and SCI group (n = 30). All animals were anaesthetized by intraperitoneal injection of ketamine (75 mg/kg) and xylazine (10 mg/kg). The back was shaved and sterilized, and an incision was made on the back posterior to the lower thoracic region. After the back muscles were infiltrated, the dorsal surface of the spinal cord was exposed by laminectomy at the T_10-12_ level, and the lower thoracic cord was subsequently transected with fine scissors. Finally, the surgical wound was closed in two layers. All SHAM rats underwent a similar operation to those in the SCI group, except that the lower thoracic cord was exposed but not transected. SCI rats received daily assistance in bladder emptying until spontaneous miction recovered. All rats were fed with commercial rat chow available ad libitum with 0.95% calcium and 0.67% phosphate, and housed in a controlled environment at 22 degree Celsius with a 12-hour light/dark cycle.

### Experimental design

Three weeks after surgery, 10 SCI and 10 SHAM rats were fasted for six hours and then euthanized. Left tibiae were immediately removed, freed from soft tissue, ten for real-time PCR analysis. Right tibiae of 10 rats per group were collected for Western blot and Immunohistochemical analysis, and the bilateral femora of per group were for harvesting marrow for cultures of the BMSC. Three months and 6 months after surgery, 10 SCI and 10 SHAM rats were sacrificed as described above, respectively. The right tibiae were obtained for Western blot analysis. To quantify whole bone mRNA and protein and to harvest marrow for cultures of the BMSC, bones, ten per group, were prepared as described below. Briefly, after death, bilateral femora and tibiae were quickly excised and soft tissues were removed. For whole bone mRNA, the epiphyses of left tibiae were removed with a razor blade, discarded, and the marrow was flushed out with a calcium- and magnesium-free PBS (PBS-CMF) solution. The metaphyses of tibiae were then flash-frozen in liquid nitrogen and stored at –70 degree Celsius before pulverization with a liquid nitrogen-cooled steel mortar and pestle and RNA isolation and protein extraction. To harvest the BMSC, bone marrow of bilateral femora were obtained for primary cultures of BMSC. The BMSC were isolated from bone marrow as described below. The medullary cavities of bilateral femora of 3-week-SCI rats were washed with DMEM-complete culture solution (GIBCO) to collect cell suspension. The expression of NK1R in BMSC-OB was detected with Western blot analysis, real-time PCR analysis at 1 week, 2 weeks, 3 weeks interval, respectively, and immunocytochemical method at 1 week. The effects of SP on the proliferation, differentiation and mineralization of BMSC-OB were observed at different concentrations and time points. We also detected the effect of SP on mRNA and protein levels of RANKL/OPG as well as on those of early and late osteoblastic markers such as type I collage (Collα1), alkaline phosphatase (ALP) and osteocalcin (OC).

### Cultivation of BMSC-OB

The cell suspension was filtered, and then centrifuged at 1000 rpm for 5 min. The cells were resuspended with standard medium and counted. The cells were inoculated in 6-well plate at 2.5×10^7^ per well, and then 50 μg of ascorbic acid, 10 mmol/L of β-glycerophosphate and 10 nmol/L dexamethasone were added to induce osteoblasts. Culture medium was changed every 3 or 4 days. After the cells underwent adherence, proliferation and passage, the cells of passage 2 were used for this experiment.

### Immunohistochemistry

The BMSC were digested, centrifuged, resuspended and counted, and then were inoculated in 6-well plate at 3.6×10^4^ (2.5 ml) per well to incubate for one weeks of osteogenic differentiation. The culture medium was changed every 3 days. Cells were fixed with 4% paraformaldehyde at 4°C for 5 min when they completely covered the walls of well. H_2_O_2_ (0.3%) was added at room temperature for 30 min and then 1% of bovine serum albumin was added to incubate for 15 min. The monoclonal antibody of rabbit anti-rat NK1R (US Biological, Swampscott, Massachusetts, USA) was diluted to 1:1000 with 0.1M PBS containing 1% of bovine serum albumin and 0.05% of sodium azide, and then was added to incubate at 4°C for 24 h. After the cells were washed three times, the secondary antibody of biotinylated goat anti-rabbit IgG (Changdao Co, Shanghai, China) was diluted to 1:200 with PBS, and then was added to incubate at 37°C for 30 min. After the cells were washed three times again, ABC (Changdao Co, Shanghai, China) was diluted to 1:100 with PBS, and then was added to incubate at room temperature for 4 h. The cells were stained with DAB for 5min. A droplet of protein glycerol was placed on a glass slide, and then the side of the cells was attached to protein glycerol. Positive expression was observed and the rate of cell-positive expression was calculated. After decalcification, the tissue specimens of right tibiae were rinsed in 20% sucrose in 0.1 M PB overnight and sectioned at a thickness of 15–30 μm on a cryostat, and then were mounted on gelatin-coated slides. The sections were processed immunohistochemically to demonstrate the presence of NK1R by means of the method as described above. The positive staining of NK1R in bone metaphyses was visible as brown punctums and bundles which were distributed in the periosteum, and bone trabeculae. We used bone marrow tissues as a positive control that showed positive brown staining and the samples without primary antibody as a negative control without positive signals. Quantitative image analyses of immunostaining intensity of NK1R from SHAM and SCI groups were performed in spongiosa of rat tibiae. It is important to emphasize that all the sections were performed at the same time for the quantitative study. The images obtained were analyzed for positive staining at a magnification of 400× using quantitative immunohistochemical analysis software Image Pro Plus (Media Cybernetics, Baltimore, MD, USA).

### Western blot analysis

Primary and secondary antibodies for NK1R (1:1000 dilution), OPG (1:500 dilution) and RANKL (1:1000 dilution) were all obtained from Santa Cruz Biotechnology. The right proximal tibial metaphysis (3–4 mm, 42.1–60.3 mg) from SCI and Sham groups were immediately weighed and stored at -80°C. Partial samples were grinded into scraps in liquid nitrogen before extraction of total protein. The expression of NK1R, OPG and RANKL in bone microenvironment was detected with Western blot analysis. The BMSC-OB of passage 2 were digested, centrifuged, resuspended and counted, and then were inoculated in 6-well plate at 3.6×10^4^ (2.5 ml) per well. In experimental group, 10^−8^ M of SP was added to incubate for three weeks, while in control group, no treatment was done. The culture medium was changed every 3 days. The cells were collected to extract total protein. After determination of protein concentration, the expression of NK1R, OPG and RANKL was detected with Western blot analysis. The cell lysate was prepared in radioimmune precipitation assay buffer containing a protease inhibitor mixture and a phosphatase mixture. The protein content was measured by the Bradford method, and equal amounts of protein were loaded for electrophoresis on a 4–20% gradient gel. The standard Western analysis protocol was used thereafter. The protein expression was detected by chemiluminescence, quantified by densitometry, and normalized to β-actin. Band intensities were quantified using Biomax 1D image analysis software (Kodak Scientific Imaging Systems).

### PCR analysis

The proximal tibial metaphysis were crushed under liquid nitrogen conditions using a Bessman Tissue Pulverizer and RNA extracted using the method of Chomczynski and Sacchi as previously described [23]. RNA integrity was verified by formaldehyde-agarose gel electrophoresis. Synthesis of cDNA was performed by reverse transcription with 2 μg of total RNA using the Superscript II kit with oligo dT(_12–18_) primers as described by the manufacturer (Sheng-Gong Technologies, Shanghai, China). cDNA (1 μl) was amplified by PCR in a final volume of 25 μl using the iQ SYBR Green Supermix (Bio-Rad, Hercules, CA, USA) with 10 pmol of each primer (Sheng-Gong). The primers of RANKL, OPG, Collα1, OC, ALP, NR1R and β-actin were listed in Table 1. RANKL, OPG, Collα1, OC and ALP gene expression in bone tissue was evaluated. In SCI group, the BMSC-OB of passage 2 were inoculated in 6-well plate at 3.6×10^4^ per well. In experimental group, 10^−8^ M of SP was added to incubate for 72 h, while in control group, no treatment was done. RANKL, OPG, Collα1, OC and ALP gene expression in BMSC-OB was determined. Real-time PCR was carried out for 40 cycles using the iCycler (Bio-Rad, Hercules, CA, USA) and data were evaluated using the iCycler software. Each cycle consisted of 95°C for 15 seconds, 60°C for 30 seconds and 72°C for 30 seconds. RNA-free samples, a negative control, did not produce amplicons. Melting curve and gel analyses were used to verify single products of the appropriate base pair size.

**Table 1:** Primer sequences used for cDNA amplication.

Gene	Primer sequence (5'-3') sense/antisense	Product size (bp)	Gene ID
ALP	GTGGTGGACGGTGAACGGGAGAA	159	NM_013059
	ATGGACGCCGTGAAGCAGGTGAG		
OC	GCAGCTTCAGCTTTGGCTACTCT	114	M25490
	CAACCGTTCCTCATCTGGACTTTA		
Collα1	GAGCGGAGAGTACTGGATCG	158	NM_007742
	GCTTCTTTTCCTTGGGGTTC		
OPG	ACAATGAACAAGTGGCTGTGCTG	109	U94330
	CGGTTTCTGGGTCATAATGCAAG		
RANKL	GCAGCATCGCTCTGTTCCTGTA	164	NM_057149
	GCATGAGTCAGGTAGTGCTTCTGTG		
NK1R	AGGACAGTGACCAATTATTTCCTGG	156	J05097
	CTGCTGGATGAACTTCTT		
β-actin	CCTGTACGCCAACACAGTGC	308	NM_017008
	ATACTCCTGCTTGCTGATCC		

### Cell proliferation

Proliferation rates were measured to analyze the dose, time-dependent manner and NK1R specificity in the effect of SP on the grow of BMSC-OB. The BMSC-OB of passage 2 were digested, centrifuged, resuspended and counted, and then were inoculated in 96-well plate at 2×10^3^ (200 μl) per well. Time-dependent manner: MTT method (Roche, Germany) for cell proliferation was performed on 24-, 48-, 72- and 96-hours-cultured BMSC-OB with 10^−8^ M SP. Dose-dependent manner: 10^−12^ M, 10^−10^ M, 10^−8^ M, 10^−7^ M and 10^−6^ M of SP were added and cultured for 96 hours. Receptor specificity: cells were cultured with 10^−8^ M of SP, 10^−6^ M of Spantide (SP antagonists, Sigma), 10^−6^ M of L703606 (NK1R antagonists, Sigma) and 10^−8^ M SP + 10^−6^ M Spantide or 10^−6^ M L703606 [24]. Proliferation was determined by MTT analysis according to manufacturer's instruction.

### Alkaline phosphatase activities

The differentiation of BMSC-OB was observed by determining alkaline phosphatase activities. The BMSC-OB of passage 2 was inoculated in 6-well plate at 3.6×10^4^ (2.5 ml) per well. Time-dependent manner: ALP activity was performed on 1-, 2-, and 3-week-cultured BMSC-OB with 10^−8^ M SP. Dose-dependent manner: 10^−12^ M, 10^−10^ M, 10^−8^ M, 10^−7^ M and 10^−6^ M of SP were added and cultured for 3 weeks. Receptor specificity: cells were cultured with 10^−8^ M of SP, 10^−6^ M of Spantide, 10^−6^ M of L703606 and 10^−8^ M SP + 10^−6^ M Spantide or 10^−6^ M L703606. The culture solution was changed every 3 days. Cells were scraped in 10 mM Tris-HCl buffer (pH 7.6) containing 10 mM MgCl2 and 0.1% Triton X-100. ALP activity was determined colorimetrically using a Sigma kit with p-nitrophenylphosphate as a substrate. The protein content was measured using a protein assay kit (Bio-Rad Laboratories, Munich, Germany) based on the Bradford method and BSA was used as the protein standard. The overall enzyme activity was expressed as units/mg protein.

### Alizarin red stain

Osteoblastic mineralization was evaluated by alizarin red stain to analyze the dose and time-dependent manner and receptor specificity in the effect of SP on the mineralization of BMSC-OB. The BMSC-OB of passage 2 were inoculated in 6-well plate at 3.6×10^4^ (2.5 ml) per well. Time-dependent manner: alizarin red stain was performed on 1-, 2-, and 3-week-cultured BMSC-OB with 10^−8^ M SP. Dose-dependent manner: 10^−12^ M, 10^−10^ M, 10^−8^ M, 10^−7^ M and 10^−6^ M of SP were added and cultured for 3 weeks. Receptor specificity: cells were cultured with 10^−8^ M of SP, 10^−6^ M of Spantide, 10^−6^ M of L703606 and 10^−8^ M SP + 10^−6^ M Spantide or 10^−6^ M L703606. The mineralized nodules measured using Alizarin Red staining. Mean number of mineralized nodules was calculated from six different fields.

### Statistical analysis

All data were indicated with (mean ± SD). One or two-factor analysis of variance with Fisher’s least significant difference (LSD) test of variance was used for the comparison between groups. Different treatment conditions were regarded as univariate factors. Student-Newman-Keuls test was used for pairwise comparisons of data. All statistical analysis was performed with SPSS11.5 software (SPSS Inc, Chicago, IL, USA). Statistical significance was established at *P* <0.05.

## Results

### Expression of NK1R in BMSC-OB

**Fig 1:**
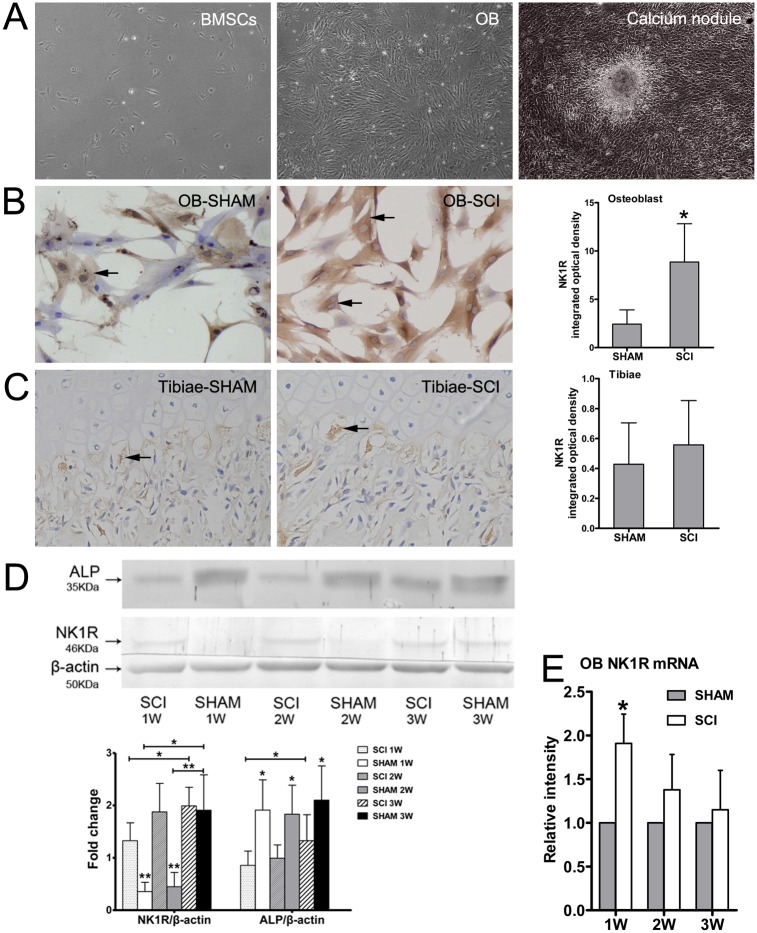
The culture of BMSC-OB and the expression of NK1R. (A) The osteobalsts derived from the second generation of BMSC after inoculation, the calcium deposition was observed at 3 weeks, x100. (B) The expression of NK1R in BMSC-OB was observed on plasma membranes and in cytoplasm (x400), and quantitative comparison of NK1R immunostaining in BMSC-OB at 1 week. (C) NK1R of the proximal metaphysis of the rat tibiae was observed at 3 weeks after surgery (x400), and quantitative comparison of NK1R immunostaining. (D) Protein expression levels of NK1R and ALP in BMSC-OB at 1 week, 2 weeks and 3 weeks after inoculation. The values of expression levels were pooled from 10 rats per group and expressed as mean ± SD. (E) Real-time PCR for NK1R expression on BMSC-OB at 1 week, 2 weeks and 3 weeks after inoculation, *p<0.05; **p<0.001.

The BMSC-OB showed adherence on the second day after inoculation. The adherent cells grew together and continuously proliferated. 3 weeks after incubation, the calcium deposition was observed (Fig 1A). Immunocytochemistry result indicated that NK1R was expressed in cell membrane and cytoplasm of BMSC-OB, especially in the cytoplasm. A significant increase of immunopositive reactions for NK1R was observed in BMSC-OB after SCI compared with SHAM ones at 1 week (Fig 1B). However, the expression of NK1R in tibiae between SCI and SHAM rats 3 weeks after surgery showed no significant differences (Fig 1C and S1 Fig). To confirm our findings regarding NK1R expression, we performed Western blot analysis using the antibody against NK1R and PCR experiment by specific primers, the results showed that the expression of both the protein and mRNA levels were in consistent with the immunoreactivity at 1 week, but this discrepancy disappeared gradually at 2 weeks and 3 weeks after osteogenic differentiation (Fig 1D and 1E). On the contrary, quantitative image analyses of protein levels for ALP demonstrated a significant and steady decrease at 1 week, 2 weeks, and 3 weeks in SCI group compared with SHAM group (Fig 1D).

### Osteoblastic markers mRNA and NK1R, RANKL/OPG protein expression in the bone microenvironment of proximal tibiae

**Fig 2:**
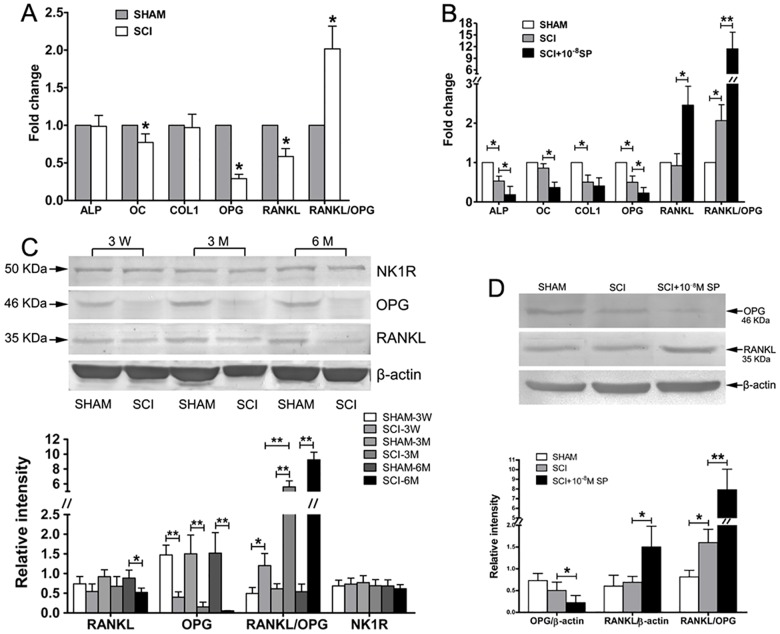
The expression of Osteoblastic markers in the bone microenvironment of proximal tibiae and the effect of 10^−8^M SP on levels of RANKL/OPG as well as on osteoblastic markers in vitro. (A) RANKL, OPG, Col1α1, ALP, and OC mRNA expression in the proximal tibiae of SCI and SHAM rats. (B) RANKL, OPG, Col1α1, ALP and OC mRNA expression in BMSC-OB treated with 10^−8^M SP. The values of expression levels were pooled from 10 rats per group and expressed as mean ± SD. (C) Western blot analysis for RANKL, OPG and NK1R proteins expression in the proximal tibiae and the expression levels were quantified by densitometry. (D) Western blot analysis for RANKL, OPG proteins expression on BMSC-OB treated with 10^−8^M SP and the expression levels were quantified by densitometry. Equal loading of protein and transfer were assessed with β-actin expression. The values of expression levels were pooled from 10 rats per group and expressed as mean ± SD. All blots were visualized by chemiluminescence, *p<0.05; **p<0.001.

The expression levels of RANKL/OPG, Collα1, OC and ALP gene in the bone microenvironment of proximal tibiae were analyzed by Real-time PCR. The results showed that the expressions of RANKL, OPG and OC in tibiae from SCI rats were significant lower than SHAM rats, but the expression of Collα1 and ALP showed no statistical significance between both groups (Fig 2A). In addition, a significant decreased expression of RANKL mRNA was observed, coupled with a more significant decreased expression of OPG mRNA in tibiae from SCI rats compared with SHAM rats. Thus, the ratio of RANKL to OPG expression in tibiae from SCI rats was significantly higher than that from SHAM rats (Fig 2A).

The expression of proteins was analyzed by Western blot experiments. In the proximal tibiae after SCI, the protein levels of OPG was reduced significantly compared with SHAM ones at 3 weeks, 3 months and 6 months after surgery, and the protein levels of RANKL was only reduced at 6 months after surgery when the expression of OPG protein was decreased more significantly as the exprssion of mRNA in Fig 2A. Thus, the RANKL/OPG in tibiae from SCI rats was significantly higher than SHAM rats (Fig 2C). However, the protein levels of NK1R in tibiae showed no significant differences between both groups (Fig 2C).

### Effects of 10^−8^ M SP on mRNA and protein levels of RANKL/OPG as well as on those of early and late osteoblastic markers in vitro

Real-time PCR and Western blot analyses were performed to determine the effects of 10^−8^ M SP on the expressions of RANKL, OPG, Collα1, OC and ALP at both the mRNA and protein levels. In BMSC-OB treated with 10^−8^ M SP for 96 hours, the mRNA and protein levels of OPG were decreased, while the mRNA and protein levels of RANKL were increased. As a result, the ratio of RANKL to OPG expression in SCI rats was significantly increased (Fig 2B–2D). In addition, the expressions of ALP, RANKL/OPG, and Collα1 in BMSC-OB from SCI rats were significantly lower than SHAM rats. Similarly, in BMSC-OB treated with 10^−8^ M SP, the expression of ALP and OC was significantly decreased. However, there was no statistical significance in Collα1 between SCI group and SCI group treated with 10^−8^ M SP (Fig 2B).

### Effects of SP on the proliferation of BMSC-OB

**Fig 3:**
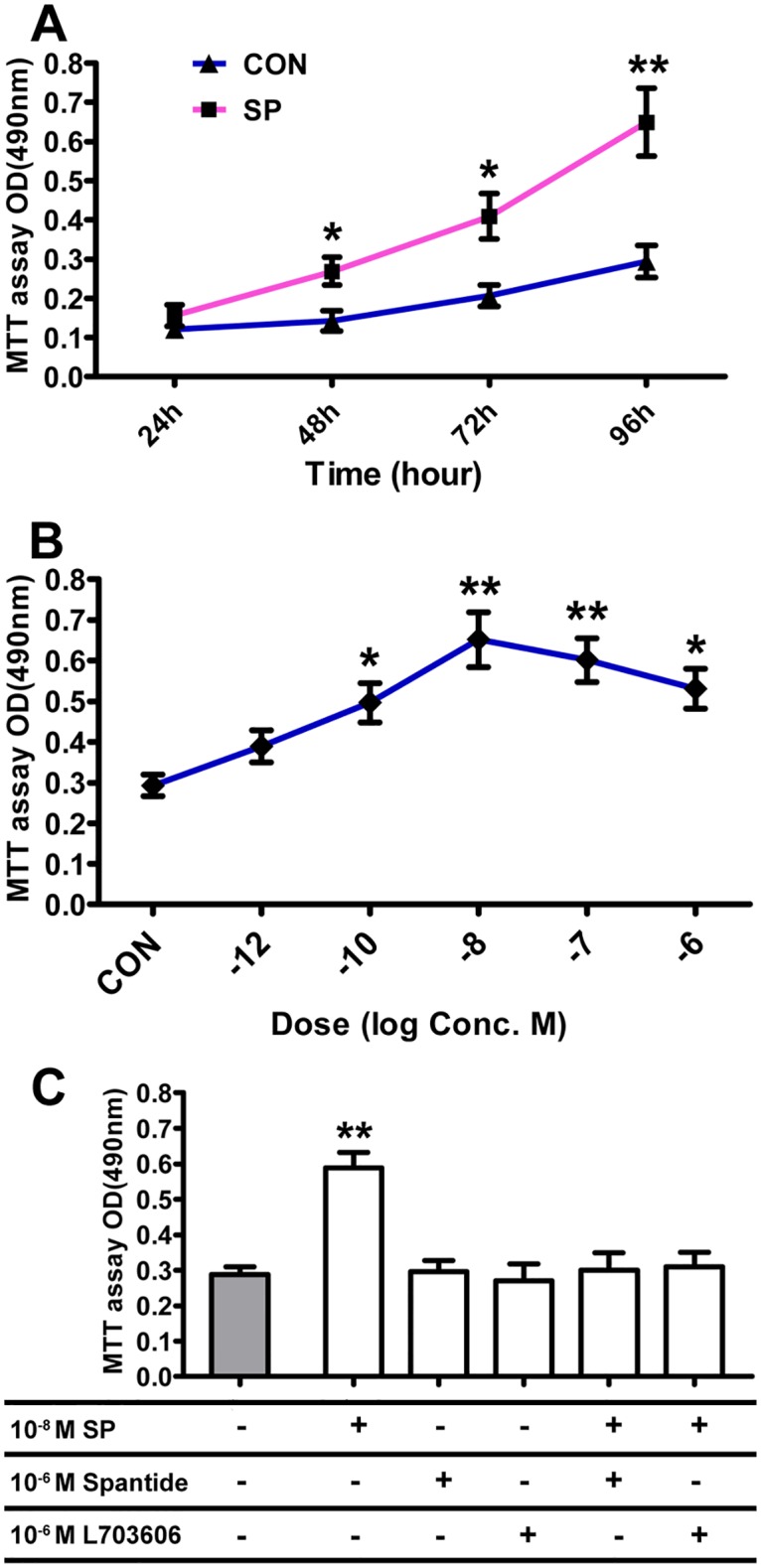
Dose- and time-dependent effects of SP treatment (10^−6^ M, 10^−7^ M, 10^−8^ M, 10^−10^ M, 10^−12^ M) on cellular proliferation by BMSC-OB after SCI. (A) The time-dependent manner of SP. BMSC-OB cultured for 96 hour with 10^−8^ M SP. Cellular proliferation increased significantly compared to the control. (B) The dose-dependent manner of SP. Cellular proliferation by BMSC-OB cultured for 96 hours without SP or with 10^−6^ M, 10^−7^ M, 10^−8^ M, 10^−10^ M and 10^−12^ M SP. (C) Effects of 10^−6^ M SP antagonist (Spantide) and 10^−6^ M NK1R antagonist (L703606) on cellular proliferation by BMSC-OB stimulated with 10^−8^ M SP. Data are mean ± SD, *p<0.05; **p<0.001.

We showed that SP could induce the proliferation of BMSC-OB at different concentrations and incubation times. BMSC-OB growth increased in response to 10^−10^ M, 10^−8^ M, 10^−7^ M and 10^−6^ M of SP at 96 hours, with the maximal effect at 10^−8^ M SP. 10^−12^ M SP also showed the promoting effect on cells proliferation, but has no significant difference (Fig 3B). 10^−8^ M SP promoted the proliferation of BMSC-OB in a time-dependent manner. NO changes of growth were observed at 24 hours, but the changes could be detected when the incubation times extendedwith the maximal changes of growth being observed at 96 hours (Fig 3A). We have studied the responses of BMSC-OB proliferation to 10^−8^ M SP alone and to SP in synergy with antagonists known to have inhibition effect on SP and NK1R. The results showed that treating 10^−8^ M SP in BMSC-OB alone resulted in an increaseof cell growth, while the effects have been inhibited by treating 10^−8^ M SP together with Spantide or L703606. The BMSC-OB treating with Spantide or L703606 alone showed no significant effect on cell proliferation (Fig 3C).

### Effects of SP on ALP activities in BMSC-OB

**Fig 4:**
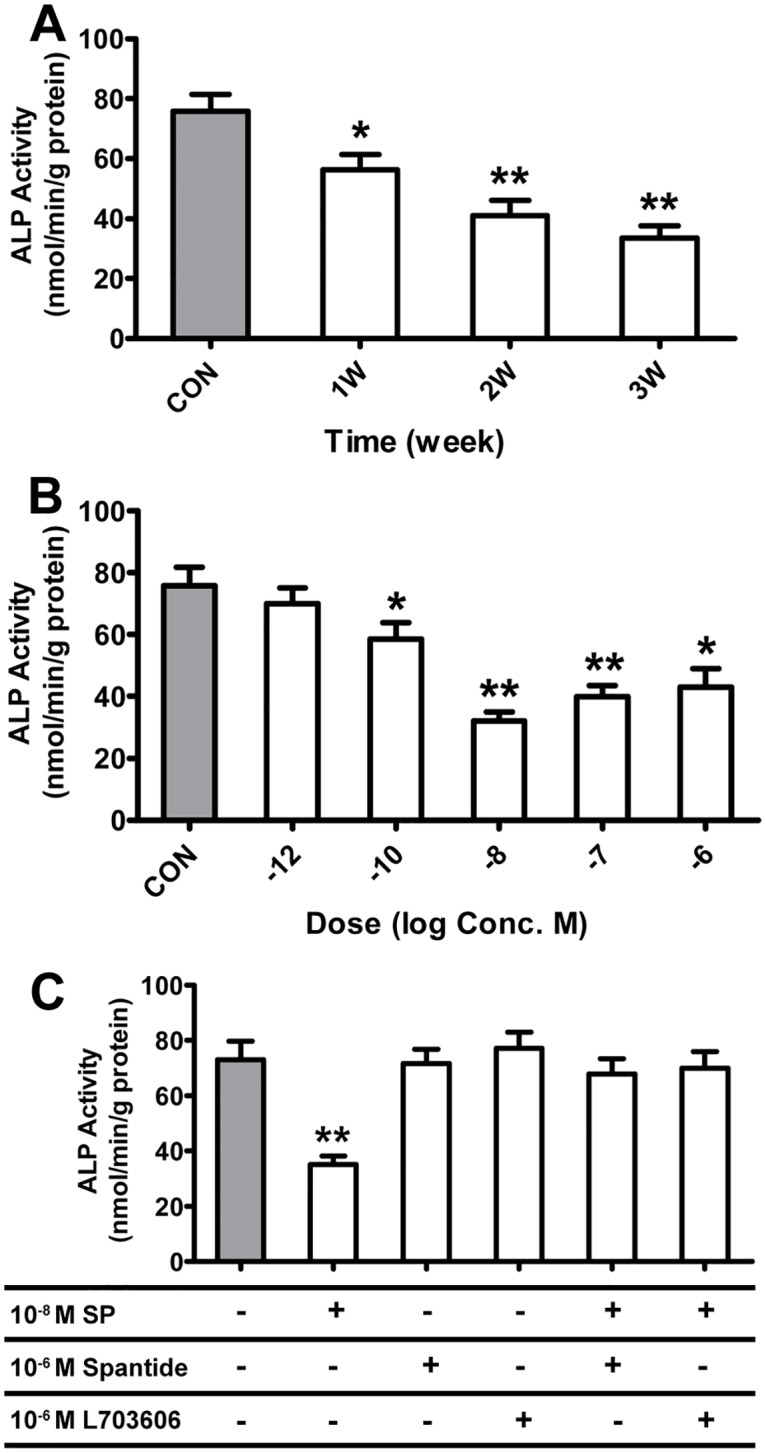
Dose- and time-dependent effects of SP treatment (10^−6^ M, 10^−7^ M, 10^−8^ M, 10^−10^ M, 10^−12^ M) on ALP activity by BMSC-OB after SCI. (A) The time-dependent manner of SP. BMSC-OB cultured for 3 weeks with 10^−8^ M SP. ALP activity decreased significantly compared to the control. (B) The dose-dependent manner of SP. ALP activity by BMSC-OB cultured for 3 weeks without SP or with 10^−6^ M, 10^−7^ M, 10^−8^ M, 10^−10^ M and 10^−12^ M SP. (C) Effects of 10^−6^ M SP antagonist (Spantide) and 10^−6^ M NK1R antagonist (L703606) on ALP activity by BMSC-OB stimulated with 10^−8^ M SP. Data are mean ± SD, *p<0.05; **p<0.001.

Treating BMSC-OB with SP at different concentrations and incubation times showed multiple effects on ALP activities. ALP activities decreased in response to 10^−10^ M, 10^−8^ M, 10^−7^ M and 10^−6^ M of SP at 3 weeks, with the maximal effect at 10^−8^ M SP. 10^−12^ M SP also had the inhibitory effect, but showed no significant difference (Fig 4B). 10^−8^ M SP inhibited ALP activities in a time-dependent manner. Significant changes were observed with longer incubation times at 1 week (S2B Fig), and the maximal changes of ALP activities were detected at 3 weeks (Fig 4A). We have studied the responses of ALP activities to 10^−8^ M SP alone or to SP in synergy with antagonists known to have inhibition effect on SP and NK1R. The results showed that treating 10^−8^ M SP alone in BMSC-OB resulted in an decrease in ALP activities, while the effects could be reversed by treating BMSC-OB with Spantide or L703606. The BMSC-OB treating with Spantide or L703606 alone had no significant effect on ALP activities (Fig 4C).

### Effects of SP on the mineralization of BMSC-OB

**Fig 5:**
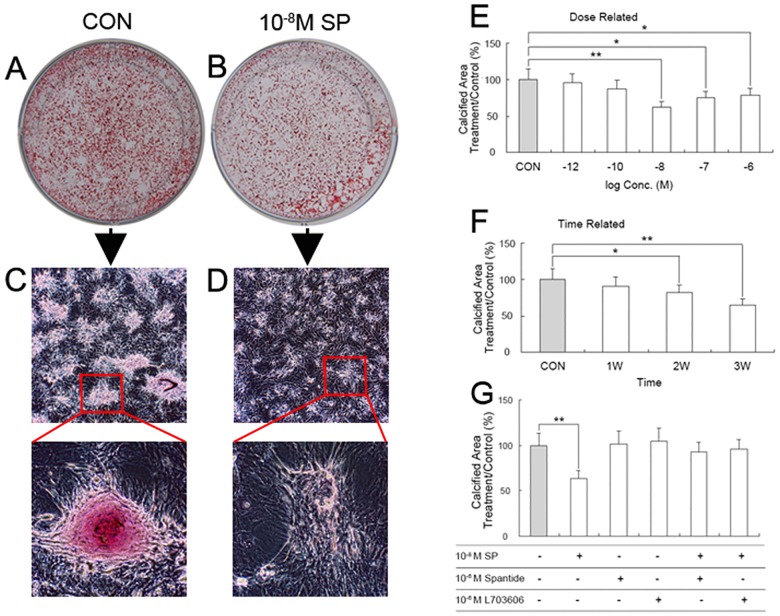
Dose- and time-dependent effects of SP treatment (10^−6^ M, 10^−7^ M, 10^−8^ M, 10^−10^ M, 10^−12^ M) on bone nodule formation in BMSC-OB after SCI. (A-B) Alizarin red stain for bone nodule formation by BMSC-OB cultured for 3 weeks without SP or with 10^−8^ M SP. (C-D) BMSC-OB formed numerous mineralized bone nodules for 3 weeks without SP or with 10^−8^ M SP (x100 & x400). (E) The dose-dependent manner of SP. Bone nodule formation by BMSC-OB cultured for 3 weeks without SP or with 10^−6^ M, 10^−7^ M, 10^−8^ M, 10^−10^ M and 10^−12^ M SP. (F) The time-dependent manner of SP. BMSC-OB cultured for 3 weeks with 10^−8^ M SP. (G) Effects of 10^−6^ M SP antagonist (Spantide) and 10^−6^ M NK1R antagonist (L703606) on bone nodule formation by BMSC-OB stimulated with 10^−8^ M SP. Data are mean ± SD, *p<0.05; **p<0.001.

Treating BMSC-OB with SP at different concentrations and incubation times showed multiple effects on mineralized nodules (Fig 5A–5D). Mineralized nodules decreased in response to 10^−8^ M, 10^−7^ M and 10^−6^ M of SP at 3 weeks, with the maximal effect at 10^−8^ M SP. 10^−12^ M and 10^−10^ M SP also had the inhibitory effect, but showed no significant difference (Fig 5E). 10^−8^ M SP inhibited mineralization in a time-dependent manner. Significant changes were observed with longer incubation times at 2 week (S2A Fig), and the maximal changes of mineralized nodules were detected at 3 weeks (Fig 5F). We have studied the responses of mineralized nodules to 10^−8^ M SP alone or to SP in synergy with antagonists known to have inhibition effect on SP and NK1R. The results showed that treating 10^−8^ M SP alone in BMSC-OB resulted in an decrease in mineralization, while the effect could be reversed by treating BMSC-OB with Spantide or L703606. The BMSC-OB treating with Spantide or L703606 alone had no significant effect on mineralization (Fig 5I).

## Discussion

SP is involved in bone formation and resorption. Previous researches illuminated that SP was capable of regulating the function of osteoclast and then inducing resorption [25, 26]. As a widely distributed neurotransmitter and neuromodulator, SP may plays an important role in bone formation of osteoblasts which needs to be further discussed [24], especially whether SP receptor is present in osteoblasts remains considerably controversial. After SCI, changes of bone microenvironment and denervation may be coordinatively involved in the activity and regeneration of the BMSC. However, it is uncertain what alteration have been made in biological characteristics of BMSC-OB and whether SP receptors are present in BMSC-OB after SCI. Our previous studies have confirmed that SP-positive nerve fibers were increased in bone tissue under the level of injury and SP content was increased in rats with SCI [21]. So far as we know, there have been a few studies indicating that the effects of SP on osteoblasts after SCI. In this study we demonstrated that NK1R is presented in BMSC-OB, and SP affects biological behaviors of osteoblasts through NK1R at the early stage of osteoblast differentiation. Furthermore, SP promotes the proliferation of BMSC-OB, but inhibits the differentiation and mineralization of BMSC-OB. SP also increases the expression of RANKL/OPG in BMSC-OB and the activities of osteoclasts, which may be one of the important causes that induce OP.

Recently, SP receptors have been detected not only in neuron and immune cells, but also in other peripheral cells, including bone cells. However, the presence of SP receptors on the osteoblast is controversial. Togar et al [27] were unable to detect SP receptor expression in human primary osteoblasts by RT-PCR. By contrast, using immunocytochemical study, Goto et al [24] recently confirmed the stimulatory effects of NK1R expression on late-stage bone formation by calvarial osteoblastic cells. In present study we demonstrated that NK1R is expressed in the cytoplasm and the cell membrane of BMSC-OB which were incubated after 1 week in both group. In addition, compared with the SHAM group, the expression of NK1R was significantly increased after SCI both at mRNA and protein levels at 1 week after osteogenic differentiation. However, it is noteworthy that this discrepancy disappeared gradually after 2 weeks, while a steady decrease of ALP protein expression was shown throughout the differentiation phase (S3 Fig). These results indicated that the expression of NK1R is associated with the early stage of osteoblast differentiation, including reducing ALP secretion due to the inhibiting effect on osteoblast recruitment and activation. Although we cannot exclude the significant difference of osteoblast differentiation at late stage between two groups, we demonstrated that the expression of NK1R has provided more information on the depression of bone formation in the early stage. On the other hand, SCI-induced sublesional bone loss in young rats at an early stage is associated with a significant increase of nerve fiber innervation density of SP-immunoreactive, thus, the changes in the expression of NK1R following SCI could be a consequence of systemic changes of SP such as its sensitivity to osteoblast at early stage. The presentation of NK1R in the BMSC-OB after SCI suggested that the growth promoting effects of SP could be direct. Therefore, we believe that NK1R expression in BMSC-OB modulates the activity of immature osteoblasts, which indicates that SP may stimulate osteoblastic activity mainly at early stage. However, the mechanism of NK1R expression following SCI at early stage needs further experimental verification.

Bone formation and resorption are coupled through the RANKL/OPG axis. Increased expression of RANKL can stimulate the recruitment and activity of osteoclast, and hence increases bone resorption [28, 29]. In this study, we detected the effect of neural lesion on the expression of RANKL/OPG in bone microenvironment and in the dynamic balance of bone formation and resorption after SCI. Our previous studies have confirmed that bone loss and bone-ultrastructural degeneration are severer in SCI-induced OP than disuse OP, and these changes are aggravated with a significant increase of nerve fiber innervation density of SP-positive nerve fibers and SP content in bone microenvironment, resulting in an increase in the osteoclastogenic potential of the denervated bone marrow over osteoblast activity [21, 22]. This study has confirmed that the expression of RANKL decreased whereas coupled with a more significant decreased expression of OPG in bone microenvironment from SCI rats compared with SHAM rats, then leading to a dramatic increase in the ratio of RANKL to OPG. We speculated that this change in bone metabolism may be associated with the increase of SP content in bone microenvironment, which directly or indirectly affects the dynamic balance of bone formation and resorption following SCI. This study has also found that 10^−8^ M SP can significantly increase RANKL expression and inhibit OPG expression in BMSC-OB, which in turn stimulates the generation and activities of osteoclasts, leading to bone resorption and bone loss. Taken together, the change of RANKL/OPG in response to SCI would be expected to stimulate osteoclastogenesis, and gradually induce bone loss. A similar effect of SP is well known to be involved in RANKL and OPG production in our study. Thus, the change in RANKL and OPG expression associated with SCI may contribute, at least in part, to the high expression of SP that occurs after the injury and the development of OP. We speculate that high expression of SP may lead to increased bone resorption through the RANKL/OPG axis after SCI. In contrast to our study, a reduced expression of serum RANKL whereas higher expression of serum OPG were observed in SCI patients in a clinical study [28]. This disassociated phenomenon is also present in postmenopausal women with OP or osteopenia [29]. This may represent a redistribution of OPG into the serum, or a compensatory response of cells other than osteoblasts to the enhanced osteoclastic bone resorption. The increased levels of OPG could bind more circulating RANKL, which may reduce the levels free RANKL after SCI.

The effects of SP on osteoblasts are also controversial, especially in the changes of denervation after SCI. SP reduced the ALP activity of rat bone-marrow-derived cells and inhibited bone nodule formation and ALP activities of rat calvarial cells [30, 31]. Meanwhile, SP was reported to stimulate osetogenesis in a dose-dependent manner [32]. In this study, we investigated the stimulatory effects of different concentration of SP on the proliferation, differentiation and mineralization of BMSC-OB after SCI. After osteoblast differentiation, BMSC express osteogenous characteristics and produce mineralized nodules and bone-like tissue in vitro [33]. After histochemical staining, microscope displayed that cells were arranged in multilayer showing spiral or stellated shapes with calcium phosphate mineralization in its central site [30]. In this study, we found that SP had variously promotive effects on BMSC-OB proliferation at different concentrations with 10^−8^ M SP the strongest one. In our MTT study, the rate of proliferation changes mainly at 48, 72, 96 hours after the stimulation with SP, and the dose of SP changes mainly at 10^−10^ M, 10^−8^ M, 10^−7^ M and 10^−6^ M, which is showing a time-dependent manner. In addition, the proliferation of BMSC-OB could be inhibited by SP antagonist (spantide) or NK1R specific antagonist (L703606). SP, an effective factor to promote mitosis, has been confirmed to induce the proliferation of many cells including endothelial cells [34], fibroblasts [35], and synovial cells [36]. Our results concur with those of other investigators who have demonstrated that SP plays a significant role in regulating the growth and proliferation of osteoblast *in vitro*, with the maximal effect at the 10^−8^ M concentration and greatest growth at 96 hours. Similar to the dose-dependent manner in this study, the responses to the concentration gradient of SP (10−12–10^−8^ M) and SP in synergy with antagonist in bone marrow-derived fibroblasts suggested that SP has a dose-related osteogenic-stimulating effect and the increase in the number and size of bone colonies by SP was most likely caused by the stimulation of stem cell mitosis, osteoprogenitor cell differentiation, or osteoblastic activity [37]. In this regard, our results with L703606 (NK-1 receptor antagonist) which inhibited SP-stimulated proliferation in a dose-dependent manner, strongly suggest that SP-mediated osteoblast mitogenic effects are NK-1 receptor-mediated.

The exact role of ALP in bone tissue has been generally considered to be involved in bone formation and calcification. In this study, we found that SP of different concentrations had variously inhibitory effects on ALP activities and cellular mineralization in time-dependent manner, and the effect was maximal at 10^-8^M SP. The inhibitory effects of SP on cell differentiation and mineralization could be reversed by SP antagonist (spantide) and NK1R specific antagonist (L703606) (S3 Fig). It has been reported that ALP activity was decreased about 17% and calcium deposits were significantly decreased about 30% in cultures treated with SP [30]. Which was in line with the data obtained from our study, SP plays a significant role in regulating ALP activities and mineralization in vitro, and the decreasing effect in response to SP was over control at 96 hours, was maximal at 10^-8^M SP. Taken together, these parameters including proliferation rate, ALP activities and mineralization, reflect different stage of development and activity of osteoblasts. Here, we demonstrated that the promotive effect of 10^−8^ M SP was maximal, which may be closer to the physiological concentration of bone microenvironment after SCI. In this study about osteoblast differentiation and mineralization after the stimulate of SP, we also used 10^−8^ M SP which is consistent with other studies [24, 37]. At the transition between proliferation and differentiation, the downregulation of genes for cell proliferation and collagen synthesis coincides with the onset of expression of previously suppressed genes for matrix maturation and mineralization.

Osteoblasts show a strongly regulated, sequential gene expression pattern, which consists of a reciprocal and functionally coupled relationship between proliferation and differentiation. Previous investigators had confirmed that SP inhibited bone nodule formation and ALP activity in a dose-dependent manner (10^−7^ M to 10^−5^ M) and further suppressed mRNA expression of bone sialoprotein, osteopontin, and osteocalcin [38]. Taken together, we infer that SP affects the development of BMSC-OB after SCI by a prolongation of cell growth and matrix production or by inhibition of cell differentiation. This hypothesis is supported by our results: SP stimulated cell proliferation and protein synthesis, but reduced ALP expression and extracellular matrix mineralization. It is worth to notice that SP has opposite effects on the proliferation and bone nodule or ALP activity with different SP concentrations. That is to say, low concentrations (10^−12^ M to 10^−8^ M) enhanced BMSC-OB proliferation but did not increase the ALP activity; high concentrations (10^−6^ M to 10^−7^ M) significantly stimulated ALP activity but did not promote the BMSC-OB proliferation. It may reflect the fact that the formation of extracellular matrix might lead to the down regulation of proliferation and the concentration of SP may give the signal to determine which direction the cells would develop into after SCI. In order to identify the specificity of SP, we used Spantide (SP antagonist) and L703606 (NK1R antagonist) in this study. Results indicate that the effects of SP on the proliferation, differentiation and mineralization of BMSC-OB may be inhibited by the two antagonists, demonstrating that SP may stimulate both the proliferation and protein synthesis of BMSC-OB when the local concentration of SP increases in a limited region and remains at a high concentration (possibly 10^−8^ M), and when the NK1R are over-expressed at early stage. This is coincided with an extensive distribution of SP-positive nerve fibers and SP content in bone tissue, so this neuropeptide may be associated with bone growth and remodeling in vivo. From the pathophysiological point of view, these findings demonstrate that the pathogenesis of OP after SCI should not simply be considered resulting from disuse, and SP itself may play an important role in the bone demineralization following SCI. The change in NK1R expression and effects of SP associated with SCI may contribute, at least in part, to the gradual loss of bone that occurs after the injury and the development of OP [21]. However, the development of OP after SCI is a complicated process involving both systemic and local changes; thus, the changes in the expression of SP and NK1R following SCI may be a consequence of systemic changes in growth factors such as insulin-like growth factor (IGF-1), leptin, and sex steroids [22].

In conclusion, besides the disuse mechanical factor, the increase of nerve injury-induced SP in bone microenvironment plays an important role in SCI-induced OP. This study has found that NK1R is present in BMSC-OB and SP affects biological behaviors of BMSC-OB through target receptor NK1R at the early stage of osteoblast differentiation. This study has also found that SP promotes the proliferation, but inhibits the differentiation and mineralization of BMSC-OB. We also demonstrated that SP increases RANKL/OPG expression of osteoblasts and activities of osteoclasts, and this may explain, in part, one of the important causes which induce OP.

## Supporting Information

S1 Fig NK1R of the trabecular bone and bone marrow of the rat tibiae was observed at 3 weeks after surgery (x400), and quantitative comparison of NK1R immunostaining. (TIF)

S2 Fig Alizarin red stain for bone nodule formation and ALP staining by BMSC-OB cultured without SP or with 10^−8^ M SP. (A) 10^−8^ M SP reduced the mineralization of BMSC-OB. (B) 10^−8^ M SP inhibited ALP activities in BMSC-OB.
(TIF)

S3 Fig Effects of 10^−6^ M SP antagonist (Spantide) and 10^−6^ M NK1R antagonist (L703606) on protein expression levels of NK1R and ALP by BMSC-OB stimulated with 10^−8^ M SP at 3 weeks after inoculation. (TIF)
