# Supplementary figures and images for: Substance P Promotes the Proliferation, but Inhibits Differentiation and Mineralization of Osteoblasts from Rats with Spinal Cord Injury via RANKL/OPG System
Source: PLoS One. 2016 Oct 20;11(10):e0165063. doi: 10.1371/journal.pone.0165063 (PMC5072631; doi:10.1371/journal.pone.0165063)

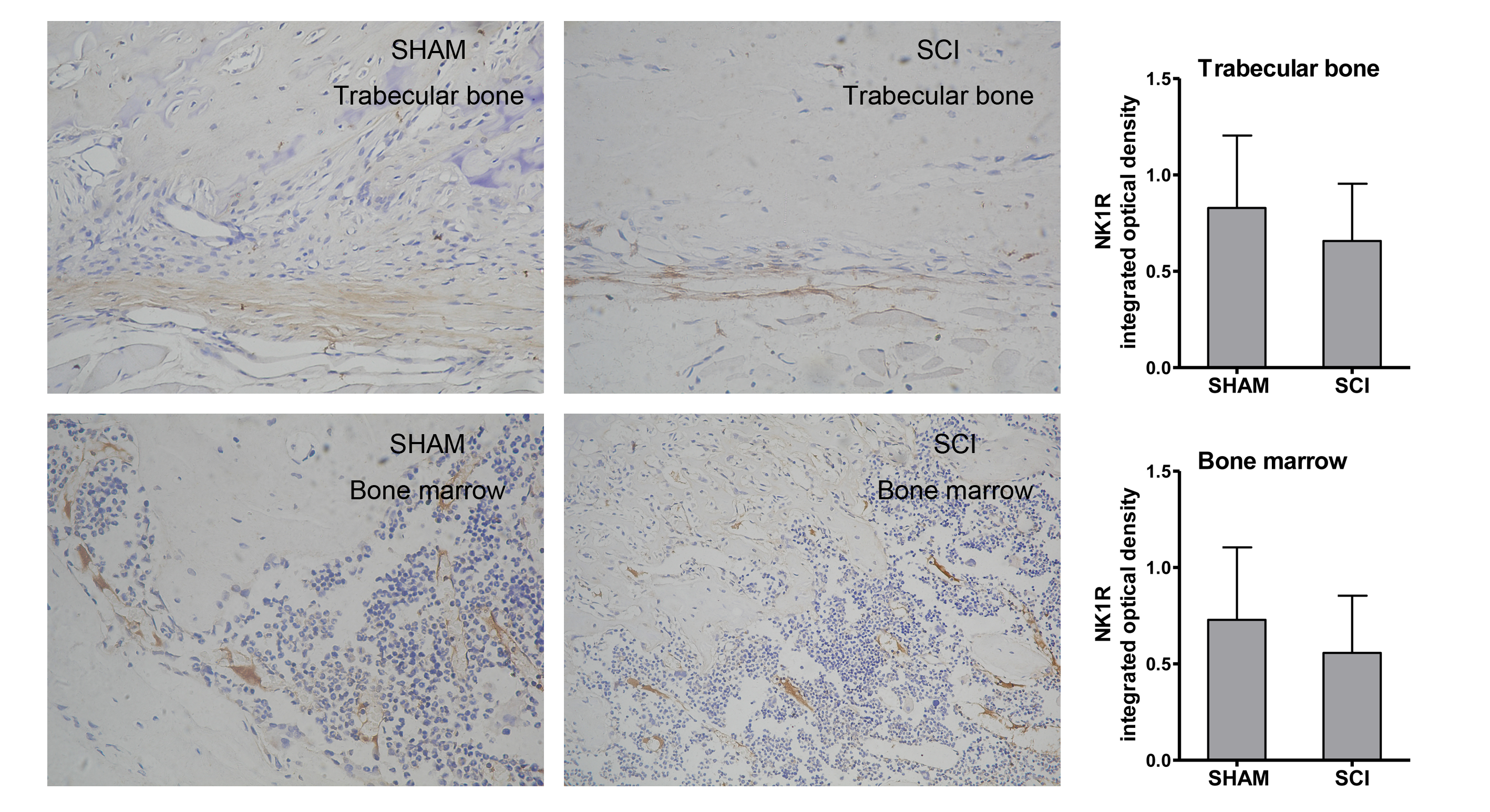

Supplement: S1 Fig — (TIF) [file pone.0165063.s001.tif]

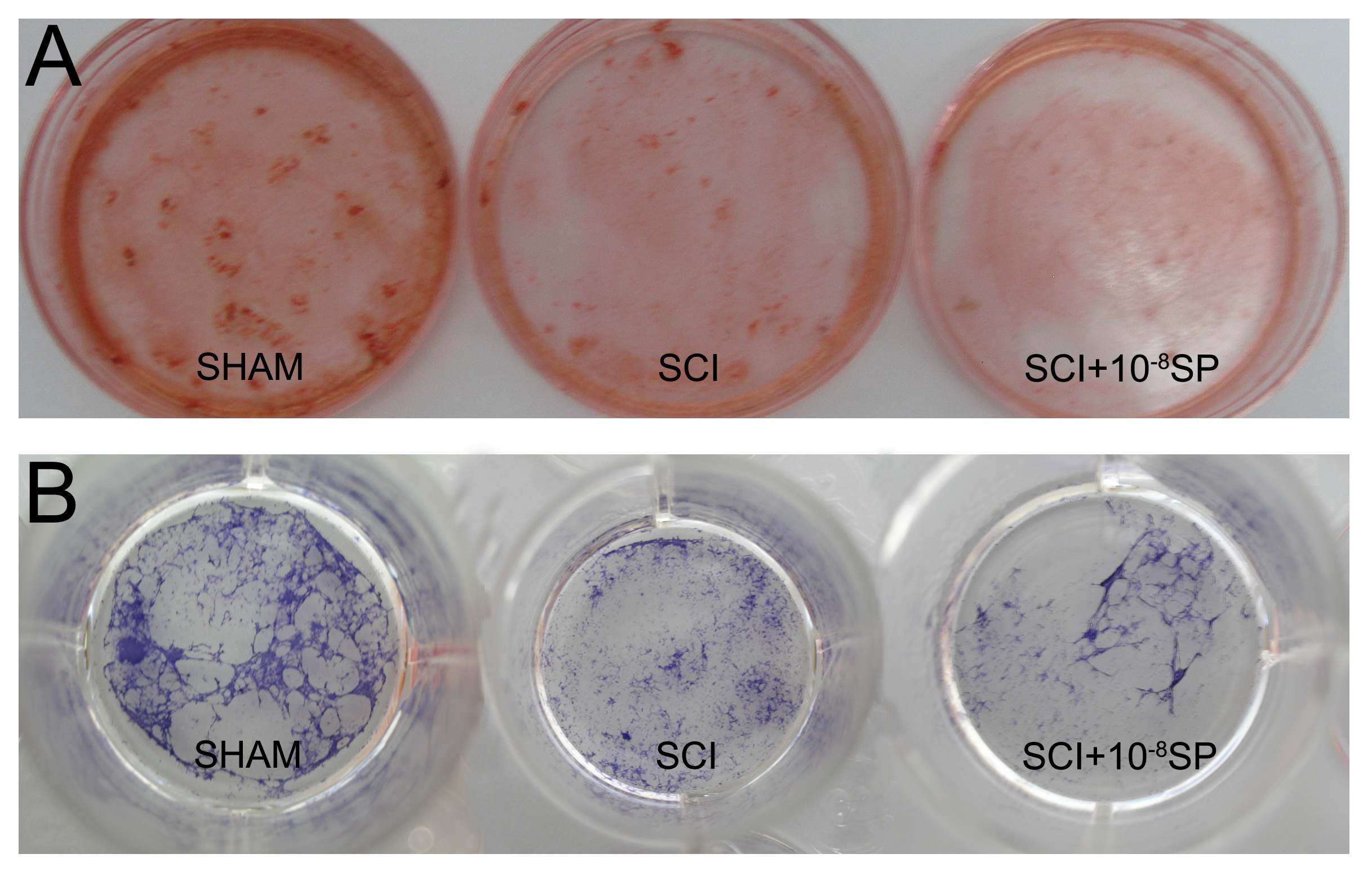

Supplement: S2 Fig — (A) 10−8 M SP reduced the mineralization of BMSC-OB. (B) 10−8 M SP inhibited ALP activities in BMSC-OB. (TIF) [file pone.0165063.s002.tif]

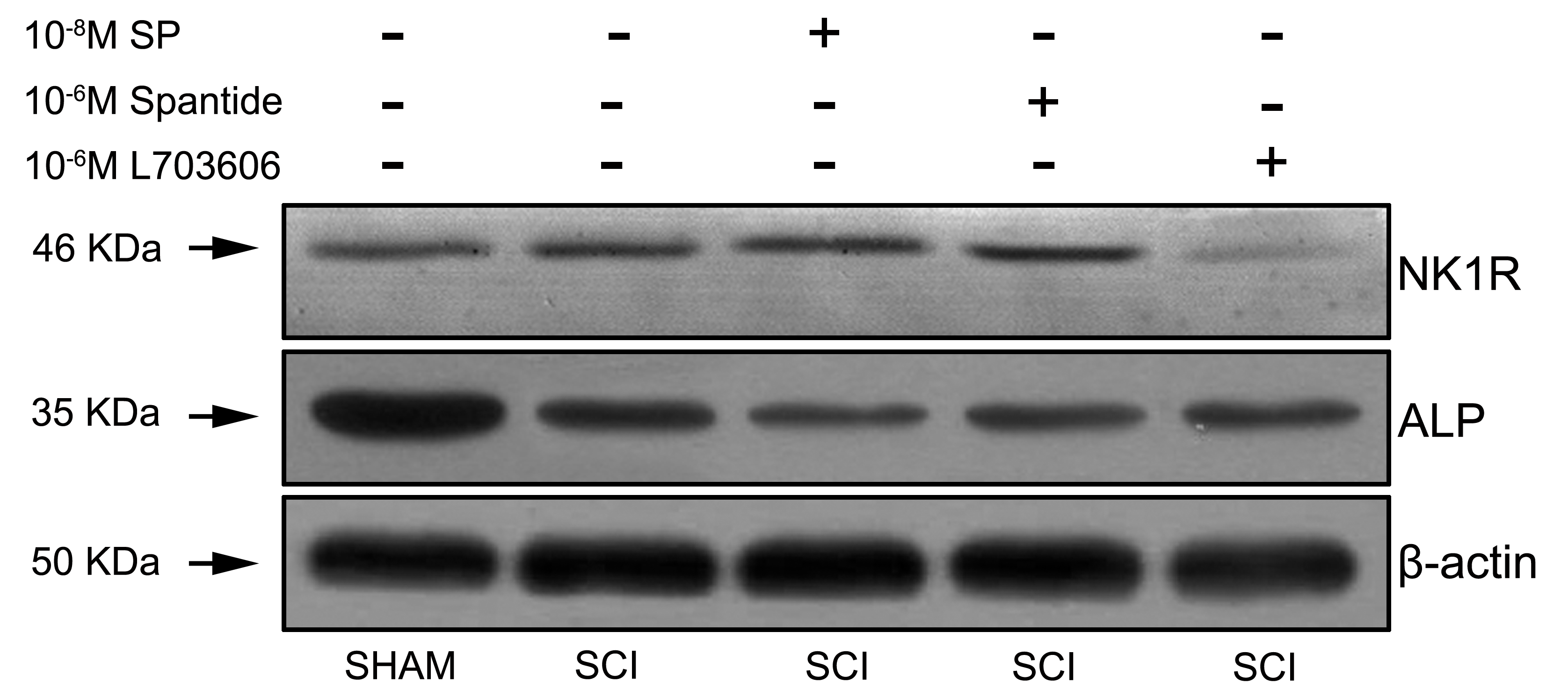

Supplement: S3 Fig — (TIF) [file pone.0165063.s003.tif]
